# CID fragmentation, H/D exchange and supermetallization of Barnase-Barstar complex

**DOI:** 10.1038/s41598-017-06507-2

**Published:** 2017-07-21

**Authors:** Yury Kostyukevich, Aleksej A. Shulga, Alexey Kononikhin, Igor Popov, Eugene Nikolaev, Sergey Deyev

**Affiliations:** 1 Skolkovo Institute of Science and Technology Novaya St., 100, Skolkovo, 143025 Russian Federation; 2 Institute for Energy Problems of Chemical Physics, Russian Academy of Sciences, Leninskij pr. 38, k.2, 119334 Moscow, Russia; 3 Emanuel Institute for Biochemical Physics, Russian Academy of Sciences Kosygina st. 4, 119334 Moscow, Russia; 4 Moscow Institute of Physics and Technology, 141700 Dolgoprudnyi, Moscow Region Russia; 5 Shemyakin & Ovchinnikov Institute of Bioorganic Chemistry of the Russian Academy of Sciences, 16/10, Miklukho-Maklaya str., Moscow, 117997 Russian Federation; 6 National Research Tomsk Polytechnic University, 30, av. Lenina, Tomsk, 634050 Russia

## Abstract

The barnase-barstar complex is one of the most stable protein-protein complexes and has a very wide range of possible applications. Here we report the use of top-down mass spectrometry for the investigation of the structure of this complex, its ionization via ESI, isolation and fragmentation. It was found that the asymmetry of the resulting charge state distributions of the protein monomer product ions increased as the charge state of the precursor ions increased. For the investigation of the 3D structure of the complex, the gas phase H/D exchange reaction was used. In addition, supermetallized ions of the complex with Zn were produced and investigated. It was observed that an increase in the number of metals bound to the complex results in a change in complex stability and the charge distribution between protein fragment. Analysis of the fragmentation pattern of the supermetallized complex [bn-b* + 5Zn]^10+^ indicated that this ion is present in different conformations with different charges and Zn distributions. Since Zn cannot migrate, such structures must be formed during ionization.

## Introduction

Ribonuclease barnase (bn, 12 kDa) and its inhibitor barstar (b*, 10 kDa) are two small soluble proteins produced by bacterium *Bacillus amyloliquefaciens*. They form a stable complex with a dissociation constant K_d_ of the order of 10^−14^–10^−13^ M, in which the active site of the enzyme is hidden and an access to the substrate is blocked^1–6^. This high-affinity molecular pair provides a convenient and flexible modular base for the design of molecular probes and protein/antigen assays^7^, a bioengineering method for self-assembly of multifunctional superstructures with in-advance programmable properties^3^, and the way for versatile, easy-to-use bioconjugation of nanomaterials^8, 9^. One useful feature of the use of proteinaceous “molecular glue” is the opportunity to attach any protein in its functional form to the key components barnase and barstar by methods of genetic engineering to construct recognition, visualization, or cytotoxic modules^10^. Its assembly with nanoparticles provides an easy and convenient way to create desirable multifunctional superstructures^3, 10^. Using this approach, it was demonstrated that barnase and barstar-assisted nanoparticle self-assembly systems have a significant resistance to high concentrations of chaotropic agents (urea and Guanidinium chloride), as well as high temperatures^11^. Detailed characterization of the barstar-barnase complex will facilitate further development of nanoparticles with biocomputing capabilities, which could be used to create sophisticated devices with a variety of biomedical applications, including intelligent sensors and theranostic agents^12^.

Barnase is a guanine-preferential endo-ribonuclease. It consists of a single polypeptide chain of 110 amino acids, which forms two N-terminal α-helices and a five-stranded anti-parallel β-sheet^13, 14^. The main hydrophobic core is formed by the packing of the first α-helix against the β-sheet. On the other side of the β-sheet a broad shallow groove runs along almost the entire length of the molecule. Residues His-102, Glu-73 and Arg-87, which are strictly conserved within the family of ribonucleases, are located in the shallow groove and form the active site. A conserved loop formed by residues 56–69 lies at one end of the broad groove and is involved in binding the guanine base of the substrate. Barstar consists of a single polypeptide chain of 89 amino acids^15^. The 3D structure of the barnase-barstar complex was investigated using X-ray^16^, NMR^15^, computer simulation^17^ and mass spectrometry^18^.

Mass spectrometry combined with ESI or MALDI ionization is a very powerful method for the investigation of non-covalent protein-ligand complexes^19^, which was used to characterize the barnase-barstar complex^18^. In order to observe gas phase ions of the protein-protein complex, “native” conditions of the experiment should be maintained^20–22^. Such requirements impose certain limitations on the use of the conventional H/D exchange approach for mass spectrometric investigations of the 3D structures of complexes^23, 24^. The conventional H/D exchange is a bottom-up technique, which consists of protein exposition in deuterated solvent, reaction quenching by lowering pH to 2.6, enzymatic digestion, and LC-MS analysis. This approach requires many steps and is sensitive to experimental conditions. Since special solution composition or pH must be maintained, it may be difficult or even impossible to use this method in an MS experiment. Top-down H/D exchange^25^, on the other hand, especially when it is performed after ionization in the gas phase^26, 27^, lacks these disadvantages. Once the complex is ionized and the 3D structure of the ion is likely to be as close as possible to obtain to the structure in the solution, then the H/D exchange reaction in the gas phase labels the outer surface of the complex and the following MS^n^ experiments reveal the exchange-protected sites. Recently it was suggested to perform the H/D exchange reaction directly in the ESI source^28, 29^. This approach was applied to study proteins^30–32^, oligosaccharides^33^, natural complex mixtures^34–37^ and small organic molecules^38^.

Here we attempt to apply the gas phase H/D exchange approach combined with collision induced fragmentation (CID) in order to get insights into the high order structure of the bn-b* complex ion in the gas phase. One of the advantageous features of the gas phase H/D exchange reaction is the use of elevated temperatures in the region of droplet evaporation, when several unusual gas phase reactions can take place. One of such reactions is the supermetallization^30^, which is also described in the paper. The investigation of the pathways of fragmentation of supermetallized complexes may provide structural information whic cannot be obtained in the H/D exchange experiments because the metal atoms are less labile than protons, so the effect of scrambling would be minimized^39–42^.

## Methods

### Samples and Instruments

Barstar A (Cys-40,82-Ala barstar) and barnase were purified from *Escherichia coli* strain BL21(DE3) harboring the corresponding expression plasmids. Harvested cells with barstar A were sonicated and nucleic acids were precipitated by gradual addition of polyethyleneimine to a final concentration of 0.03%. Cleared lysate was fractionated with ammonium sulfate. The protein fraction precipitated in the saturation interval 40–80% was purified on a XK 16/70 column packed with Sephadex G-50 SF (GE Healthcare). Barstar A containing fractions were pooled, diluted 6-fold with 50 mM Tris-Cl, рН 8.0, and applied on MonoQ 10/100 GL (GE Healthcare). The protein was eluted by NaCl gradient 0–1 M. Peak fractions were exhaustively dialyzed against 10 mM EDTA, pH 7.5.

**Figure 1:**
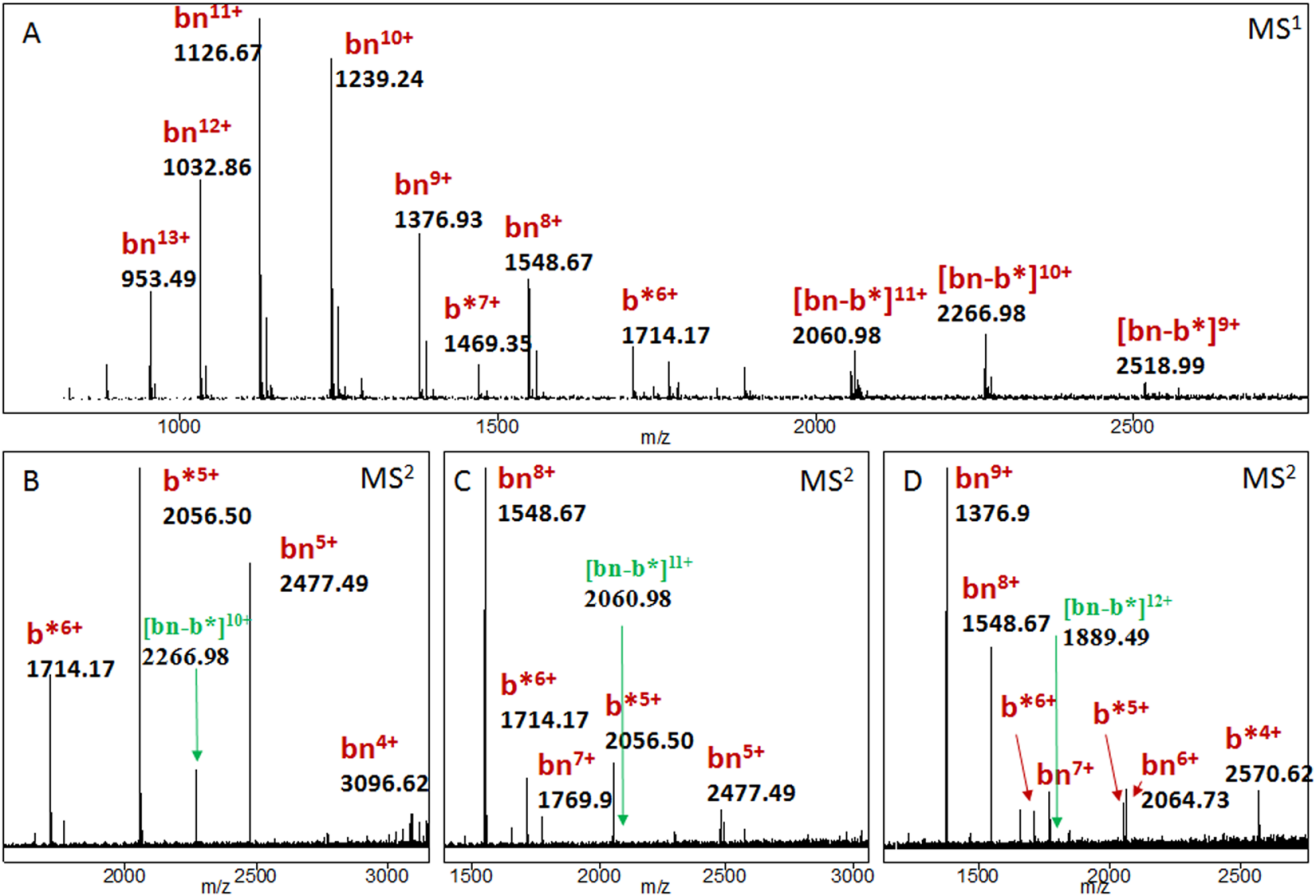
Ionization and fragmentation of the bn-b* complex. (**A**) - The broadband mass spectrum. (**B**) – Fragmentation of [bn-b*]^10+^, (**C**) –fragmentation of [bn-b*]^11+^, (**D**) –fragmentation of [bn-b*]^12+^. The precursor ions are given in green. For the full range spectrum see Figure S2.

Barnase was secreted mainly into the growth medium and then was extracted onto phosphocellulose P11 (Whatman). It was further purified by hydrophobic (Phenyl Sepharose FF) and cation-exchange (MonoS 10/100 GL) chromatography, then exhaustively dialyzed against 10 mM EDTA, pH 7.5. The resulting proteins were essentially pure in SDS-PAGE (Fig. 1). Protein concentration was determined with an Ultrospec 7000 spectrophotometer (GE Healthcare), assuming the following molar extinction coefficients at 280 nm (in M^−1^ cm^−1^): 26 030 for barnase and 20 910 for barstar А. To prove complex formation, a native electrophoresis experiment was performed as described by Laemmli^43^ and Ornstein& Davis^44^ (see Fig. S1). The gel percentage was 12%.

For mass spectrometric experiments, a solution containing 0.5 M CH_3_COONH_4_, 2% glycerol, and 0.5% m-NBA was prepared. The solutions of bn and b* in water with concentrations of 250 μM were mixed, incubated for 5 minutes at 80 °C, and diluted in the buffer solution in order to obtain a concentration of 10 μM. This final solution was directly introduced into the ESI source. For deuterium labeling, the buffer was prepared in D_2_O. D_2_O was purchased from the “Neogaz” company. Enrichment of D_2_O was 99.9%. Other chemicals were purchased from “Sigma” and were of analytical grade or higher.

### MS analysis

All experiments were performed on a LTQ FT Ultra (Thermo Electron Corp., Bremen, Germany) mass-spectrometer equipped with a 7T superconducting magnet. Ions were generated by an IonMax Electrospray ion source (Thermo Electron Corp., Bremen, Germany) in positive ESI mode. The temperature of the desolvating capillary was varied from 50 °C to 450 °C. The length of the capillary was 105 mm and its inner diameter was 0.5 mm. The infusion rate of the sample was 1 μL/min and the needle voltage was 4000 V.

### H/D exchange

For in-ESI source H/D exchange, the atmosphere was saturated with D_2_O vapors in the region between the ESI tip and the inlet of the desolvating capillary by placing 400 μL of D_2_O on a copper plate positioned approximately 7 mm below the ESI needle. More details about the used H/D exchange approach can be found elsewhere^29^. For exchange in the liquid phase, aqueous solutions of bn and b* with concentrations of 250 μM were mixed, incubated at 80 °C for 5 minutes and diluted with a deuterated buffer. Important information about the sequences of proteins and number of labile hydrogens is given in Table S1.

## Results and Discussion

### Ionization and fragmentation of the bn-b* complex

The broadband mass spectrum of the prepared solution is presented in Fig. 1. It consists of peaks corresponding to the ions of barnase with charges varying from 7+ to 14+, ions of barstar with charges varying from 9+ to 5+, and ions of bn-b* with charge states varying from 9+ to 12+. It was observed that the shape and relative intensities of the different ion peaks in the mass spectra significantly depend on the ion optics settings of the mass spectrometer. In order to observe the ions of bn-b*, the solvent composition should be as described in the “METHODS” section. With other solvents, such as H_2_O:MeOH mixtures, and different additives like formic acid, or NH_4_(HCO_3_), instead of CH_3_COONH_4_, the intensities of the complex ion peaks were very weak, if they were observed at all. We should mention that for the H_2_O:MeOH mixtures the [b*-b*]^11+^ion was observed^45^. Multiply ionized complex ions [bn-b*]^10+^, [bn-b*]^11+^, and [bn-b*]^12+^ were isolated in the ion trap and fragmented using CID (see also Figs S2 and S3). It was found that when electron capture dissociation (ECD) fragmentation was used, the ions’ charge decreased without fragmentation. The fragmentation spectra are presented in Fig. 1. It can be seen that when the ion [bn-b*]^10+^ is fragmented, it yields almost equal amounts of bn^5+^ and b*^5+^ monomeric ions and, with lower intensities, b*^6+^ and bn^4+^ ions, so the charge is split almost symmetrically. When higher charge states of the bn-b* complex are fragmented, a charge distribution asymmetry is observed: barnase monomers retain most of the charge. This can be explained by the assumption that the barnase monomer has more basic sites on its surface available for protonation. The fragmentation of [b*-b*]^11+^ produced ions b*^5+^ and b*^6+^ with almost equal intensities and also ions b*^7+^ and b*^4+^, but their intensities were considerably lower. Previously, asymmetric dissociation of the protein homodimers as a function of the charge state and the conformation of the complex was observed and investigated^46, 47^. The effect was explained by partial unfolding of one of the monomers, followed by proton migration to the unfolded one. Overall, the problem of conformational changes and correlation between the solution phase structures and gas phase structures is not yet resolved^48^; recently evidence was provided for the gas phase folding of peptides^49^.

The presence of the highly charged bn and b* ions can be explained by in-source fragmentation of the bn-b* complex. The LTQ FT mass spectrometer has a relatively hard skimmer-nozzle interface, in which non-covalent complexes may undergo fragmentation. As described many times earlier, CID fragmentation of protein complexes results in a charge rearrangement and subsequent ejection of a single highly charged subunit^50–52^.

### H/D exchange of the bn-b* complex

In order to obtain more structural information about the bn-b* complex, gas phase H/D exchange was applied. If the complex is subjected to H/D exchange in the gas-phase, then the CID fragmentation splits the complex into individual proteins with non-deuterated regions corresponding to the binding sites. Further fragmentation of these protein ions may reveal the exact localization of the protected regions. Unfortunately, neither barnase nor barstar could be fragmented with high efficiency by both CID and ECD approaches in our experimental conditions. Only the highest charge states (bn^15+^ and b*^8+^) show abundant fragments (See Figs S4 and S5). Nevertheless, it was possible to determine the dependence of the number of exchanged hydrogens from the temperature.

**Figure 2:**
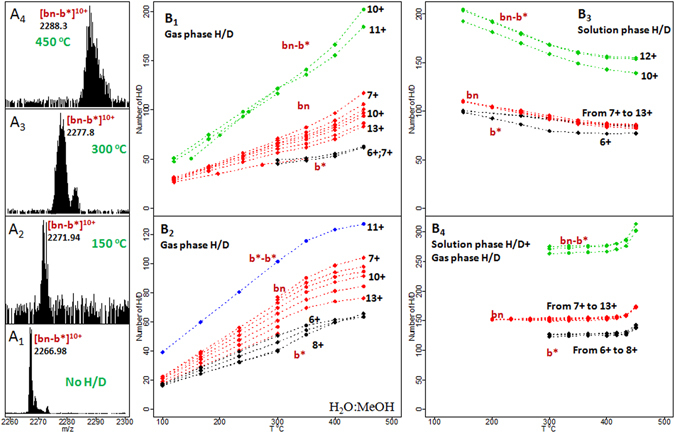
H/D exchange experiments. (**A**_**1**_–**A**_**4**_) – mass shift of [bn-b*]^10+^ as a function of the temperature of the desolvating capillary. The average m/z of the isotopic cluster is shown. (**B**_**1**_) shows the number of exchanges in the gas phase H/D exchange reaction. (**B**_**2**_) - the number of exchanges for the gas phase H/D exchange in a H_2_O:MeOH solution. (**B**_**3**_) - the number of exchanges in case of an in solution H/D exchange reaction. (**B**_**4**_) - the number of the exchanges under the combined solution plus gas phase H/D exchange conditions. Black numbers show the charge state of the corresponding ion shown in red. Lines: green – bn-b* complex, red – bn, black – b*, blue – b*-b* complex observed for a H_2_O:MeOH solution.

Mass shifts caused by H/D exchange are demonstrated in Fig. 2A_1_–A_4_. In Fig. 2B_1_,B_2_ the dependence of the number of exchanges on the temperature for the in-ESI source H/D exchange is presented. Barstar b* ions exchange almost the same number of hydrogens in all charge states at all temperatures. The maximum number of exchanges was ~62 (37% of all labile hydrogens and 79% of the fast exchangeable ones) at 450 °C.

The usage of high temperature capillary requires certain precautions, because in addition to the desirable H/D exchange reaction, ions continuously accumulate vibration energy what eventually may lead to their thermal dissociation, as described previously^53–58^. It should be specifically noted that the protein ions are not equilibrated to the temperature of the ion source.

In the case of bn, the number of exchanges decreases with the increasing charge state and for highest temperature it varies from 83 (or 40% of all exchangeable hydrogens or 78% of the fast ones) for bn^13+^ to 117 (55% of all labile hydrogens) for bn^7+^. In a bn-b* complex at 450 °C the number of exchanges is 184 (48% of all) and 202 (53% of all) hydrogens in [bn-b*]^11+^ and [bn-b*]^10+^ correspondingly. Similar results were obtained with the H_2_O:MeOH solution: the number of exchanged hydrogen atoms in [b*-b*]^11+^ complex is 127 (38% of all hydrogens in the complex).

The H/D exchange reaction was also performed in the solution phase, and after preparation the sample was immediately sprayed into the mass spectrometer. The total number of exchanges practically did not change with time but decreased with temperature because of the back exchange reaction during ESI (Fig. 2B_3_). This happens because the traces of atmospheric moisture cannot be fully removed, and as a consequence, an increase in temperature facilitates the back exchange.

To prevent this process, the atmosphere in the ESI region was saturated by D_2_O vapors. The results of the combination of the solution phase and gas phase H/D exchange methods are shown in Fig. 2B_4_. It can be seen that the infusion of D_2_O vapors prevents the back exchange reaction as anticipated. For a wide range of temperatures, the number of exchanges remains almost the same for all charge states: ~155 (73% of all) for bn, ~127 (76% of all) for b* and ~275 (72% of all) for bn-b*. However, when the temperature was increased to 450 °C, ~173 (79%) exchanges for bn, ~142 (84%) for b*, and ~310 (81%) for bn-b* were observed. This indicates that though the infusion of D_2_O initially prevents the back exchange, with an increase in the temperature additional H/D exchange occurs.

**Figure 3:**
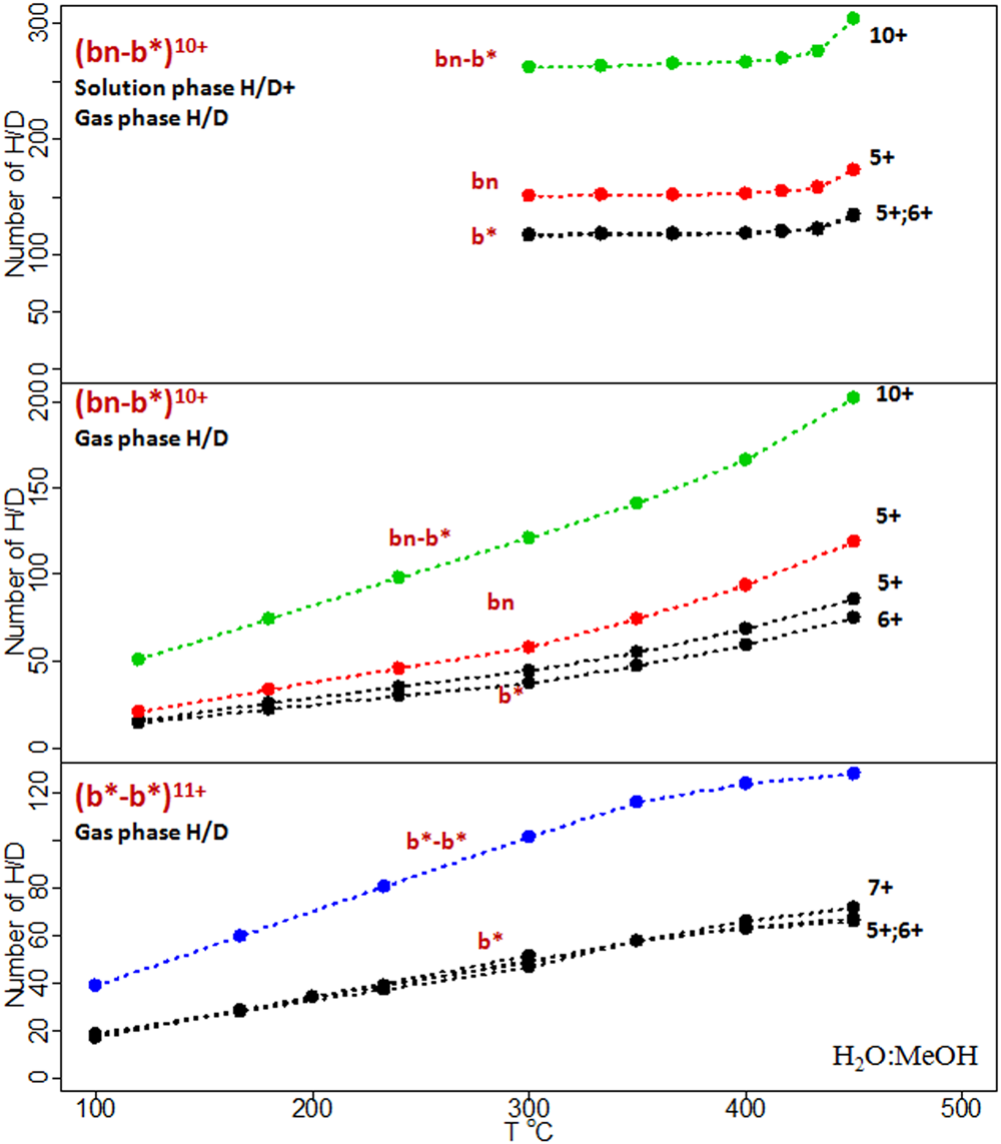
The tandem MS experiment. The numbers of exchanges in the parent ion and in the fragments are shown. Black numbers show the charge state for the corresponding ion shown in red. Lines: green – bn-b* complex, red – bn, black – b*, blue – b*-b* complex observed for a H_2_O:MeOH solution.

Further, ion complexes were isolated after the H/D exchange reaction and fragmented using CID. The results are presented in Fig. 3. When b*-b* complex is fragmented, b* ions in all charge states carried almost equal numbers of deuterium atoms, what means that this complex has a rather symmetrical structure. When the bn-b* complex is fragmented, the number of exchanges in the bn fragments exceeds the number of exchanges in the b* fragments, what is expected due to the asymmetrical nature of this complex. For the gas phase reaction at the maximum achievable temperature, 202 (53%) exchanges in the [bn-b*]^10+^ complex was observed, of which the bn^5+^ fragment showed 118 (55% of all labile hydrogens) exchanges, the b*^5+^ fragment had 86 (52% of all labile protons) exchanges and b*^6+^ demonstrated 75 (80% of fast exchangeable and 45% of all labile hydrogens) exchanges.

When the reaction was performed both in solution and in the gas phase, 304 (80% of all) exchanges was observed in [bn-b*]^10+^, 174 (81%) in fragment bn^5+^ and 135 (80%) in fragments b*^5+^ and b*^6+^. This is consistent with the data from Fig. 3B_4_, which demonstrates that when the solution phase and gas phase H/D exchange reactions are combined, there is no difference in the number of exchanges for different charge states of the same protein. The difference, when it is observed, is simply the result of the back exchange reaction.

The obtained results lead to the assumption that the [bn-b*]^10+^ ion exists in two different states: both subunits carry 5 protons each or bn carries 4 and b* carries 6. This is reasonable, since previously many researchers demonstrated, using the Ion Mobility Spectrometry (IMS)^59, 60^ or gas-phase H/D exchange^61^ or a combination of these approaches^62, 63^, that several conformers can be present within a single charge state of a protein. If two such states are present, then the deuterium distribution between the subunits should differ. Indeed, the efficiency of the gas phase H/D exchange reaction of a protein depends on its charge, so in case of a tightly folded complex, the number of exchanges in the surface region should depend on the local charge distribution.

CID of the protein complex is complicated by proton rearrangement and accompanied by H/D scrambling^39–42^. After collisional activation, protons on the surface of the complex can migrate, this results in the rearrangement of not only charge but also of deuterium. If the rate of deuterium scrambling is of the same order as the rate of the charge transfer^42^, then all conformers will redistribute the deuterium to the most favorable configuration for the given charge distribution.

In Table 1 the deuterium distribution between bn and b* subunits after the fragmentation of the [bn-b*]^12+^ complex is presented. The average number of H/D exchanges in an isolated [bn-b*]^12+^ complex is 96. The temperature was 300 °C. The fragmentation of the complex leads to the formation of subunits in different charge states. Each row in the table corresponds to an individual fragmentation channel so that the sum of charges is equal to the charge of the complex (z = 12). As can be seen, deuterium distribution on the subunits correlates with the charge distribution. These results suggest that either there is no H/D scrambling and no charge migration (otherwise the subunits with the same charge but different numbers of incorporated deuteriums would have been observed) or that the rates of these two processes are the same.

**Table 1:** Deuterium distribution between bn and b* subunits after the fragmentation of the [bn-b*]^12+^ complex.

bn		b*		Sum (z = 12)
z	Number of H/D	z	Number of H/D	
6+	59.1	6+	36.48	95.58
7+	56.56	5+	37.65	94.21
8+	52.96	4+	40.72	93.68
9+	51.39	3+	—	—

Each row corresponds to an individual fragmentation channel so that the sum of charges is equal to 12. The average number of H/D exchanges in the isolated [bn-b*]^12+^ is 96. T = 300 °C. Symbol “—” indicates that the data is not available.

### Supermetallization

Supermetallization is a recently observed phenomenon of the formation of complex peptide-metal ions in the gas phase when a peptide accommodates an unexpectedly large number of metal atoms^39, 64^. It was found that supermetallization takes place during electrospray ionization when charged droplets are evaporated under relatively high temperatures (~400 °C). The masses of supermetallized ions obey the equation:

$${{\rm{M}}}_{{\rm{complex}}}={{\rm{M}}}_{{\rm{peptide}}}+{\rm{n}}\times {{\rm{M}}}_{{\rm{Metal}}}-{{\rm{V}}}_{{\rm{Metal}}}\times {\rm{n}}\times {\rm{MH}}+{{\rm{zM}}}_{{\rm{H}}},$$

where M_complex_ is the mass of the formed complex, M_Metal_, M_H_, M_peptide_ are the masses of the metal, hydrogen, and peptide; n is the number of metal-adducts, V_Metal_ is the valence of the metal, and z is the charge.

**Figure 4:**
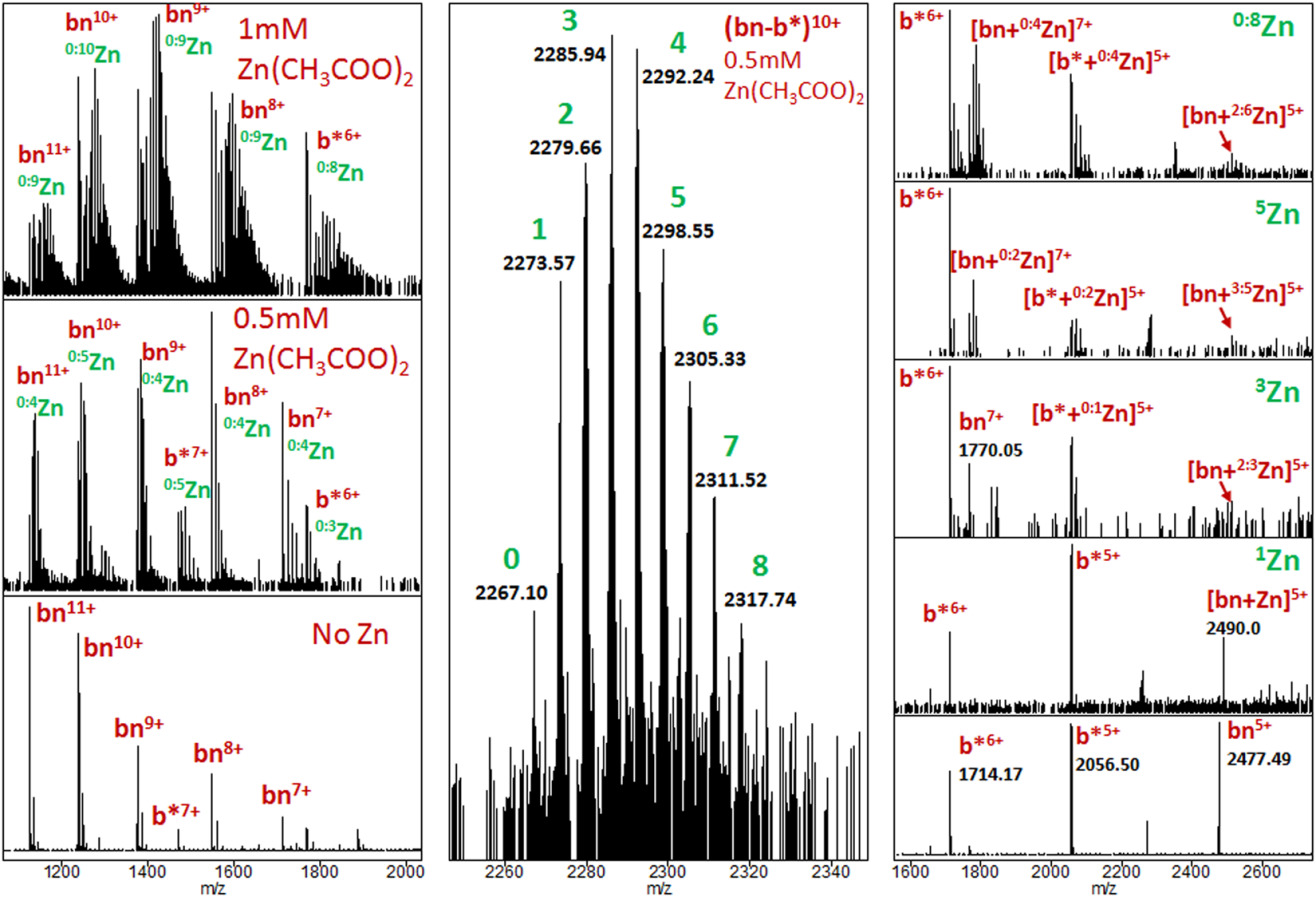
The supermetallization experiment. Temperature 450 °C. Left – the broad band mass spectrum under supermetallization conditions for different concentrations of Zn(CH_3_COO)_2_. Center – zoomed on the supermetallized [bn-b*]^10+^. This spectrum was obtained by isolating ions in the ion trap using an isolation window of 100 Da around the m/z = 2300. Right – isolation and fragmentation of the ions of [bn-b*]^10+^ carrying different numbers of Zn atoms. Green labels indicate the number of Zn atoms in the complex ion. ^x:y^Zn means that ions are carrying from x to y atoms of Zn.

In previous experiments it was shown that the most suitable solution for supermetallization is 1:1 H_2_O:MeOH with the addition of 0.1% of formic acid^34, 65^. However, the solution used in this study was different, so it was difficult to observe high intensity peaks of supermetallized ions. In order to perform the supermetallization experiments, different concentrations of Zn(CH_3_COO)_2_ were added to the buffer solution. The results are presented in Fig. 4 (see also Fig. S6 for a wider m/z range). ^x:y^Zn means that ions carry from x to y Zn atoms. As can be seen, barnase can attach up to 9 Zn atoms and barstar up to 8. The complex [bn-b*]^10+^ can carry up to 8 Zn atoms. Fragmentation of complexes carrying different numbers of Zn atoms demonstrates the asymmetry of Zn distribution between the fragments - the majority of bound Zn atoms are carried by barnase. In addition, an increase in the intensity of the bn^7+^ complexes with Zn can be seen with an increase in the number of Zn in the parent complex ion.

**Figure 5:**
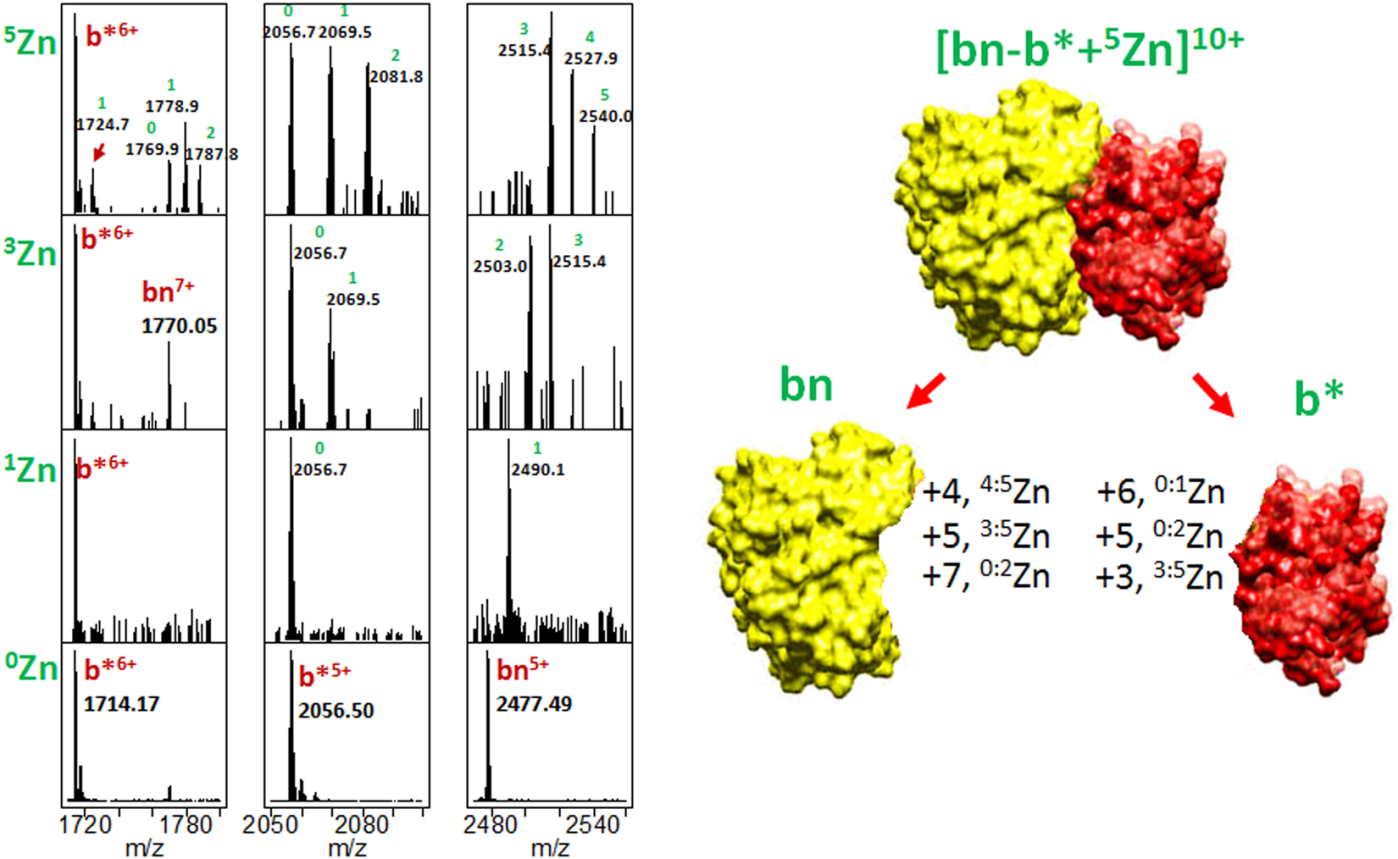
Left - zoomed parts of the fragmentation spectrum of the [bn-b*]^10+^ complex carrying different numbers of Zn atoms. Right – the correlation of charge and Zn distribution between protein monomers. ^x:y^Zn means that ions are carrying from x to y atoms of Zn.

Analysis of the charge and Zn distribution between protein monomers (Fig. 5) reveals that the number of Zn atoms attached to the protein monomer tends to increase with the decrease of its charge. Since Zn does not migrate along the protein surface^64^, since it is coordinated by several bonds, these results serve as additional evidence of the charge distribution preservation during the transition from the charged droplet to the gas phase. Based on the fact that monomeric proteins produced via CID fragmentation of complexes of higher order could not be further fragmented using ECD or CID one can conclude that the bn and b* monomers retain their folded^66, 67^ (though maybe non-native) conformation when transferred to the gas phase and metalized. This is consistent with the observation that cationization of a protein suppresses the effect of scrambling^68^.

## Conclusion

In this work the ionization and fragmentation of gas phase barnase-barstar complexes in different charge states were studied. In the in-ESI source H/D exchange reaction, bn ions demonstrated an increase in the number of possible exchanges with the decrease of the charge state. Similar effects were observed during fragmentation of the complex ions. The ion [bn-b*]^10+^ fragmented mainly to bn^5+^ and b^*5+^ giving peaks of almost equal intensity. With an increase in the total charge of the complex ion, the charge distribution among the dissociation products changes, due to an increase in the charge carried by barnase. When [bn-b*]^10+^ was fragmented, fragment b*^5+^ demonstrated fewer possible exchanges than the fragment b*^6+^. Similar results were observed for the [bn-b*]^12+^ complex. The fragmentation of free monomers revealed that only the highest charge states (bn^15+^ and b*^8+^) can produce abundant CID or ECD spectra. We also failed to fragment subunits produced after the CID dissociation of the complex [bn-b*] ion. This may be due to the internal hydrogen bonds that keep the 3D structure so that higher energy is required to induce fragmentation. Basing on the H/D exchange results we conclude that either there is no H/D scrambling and no charge migration or that the rate of of these processes is the same. In addition, an ion complex supermetallized with Zn was produced and investigated. Analysis of the fragmentation pattern of the supermetallized complexes indicates that the [bn-b*+5Zn]^10+^ ion is present in different conformations with different charge and Zn distributions. Since Zn cannot migrate, such structures must be formed during the ionization process.

## Electronic supplementary material

Supplementary Information 
