# Supplementary material for: CID fragmentation, H/D exchange and supermetallization of Barnase-Barstar complex
Source: Sci Rep. 2017 Jul 21;7:6176. doi: 10.1038/s41598-017-06507-2 (PMC5522418; doi:10.1038/s41598-017-06507-2)
Supplement: Supplementary file 1 — Supplementary Information [file 41598_2017_6507_MOESM1_ESM.pdf]

CID fragmentation, H/D exchange and supermetallization of Barnase-Barstar complex

Yury Kostyukevich[a,b,c,d], Aleksej A. Shulga[e], Alexey Kononikhin[b,d], Igor Popov[c,d], Eugene Nikolaev[a,b,c,d*], Sergey Deyev[e,f]

[a] Skolkovo Institute of Science and Technology Novaya St., 100, Skolkovo 143025 Russian Federation
[b] Institute for Energy Problems of Chemical Physics, Russian Academy of Sciences, Leninskij pr. 38, k.2, 119334 Moscow, Russia;
[c]Emanuel Institute for Biochemical Physics, Russian Academy of Sciences Kosygina st. 4, 119334 Moscow, Russia.
[d] Moscow Institute of Physics and Technology, 141700 Dolgoprudnyi, Moscow Region, Russia
[e]Shemyakin & Ovchinnikov Institute of Bioorganic Chemistry of the Russian Academy of Sciences, 16/10, Miklukho-Maklaya str., Moscow, 117997, Russian Federation
[f]National Research Tomsk Polytechnic University, 30, av. Lenina, Tomsk,634050 Russia

Supplementary Information

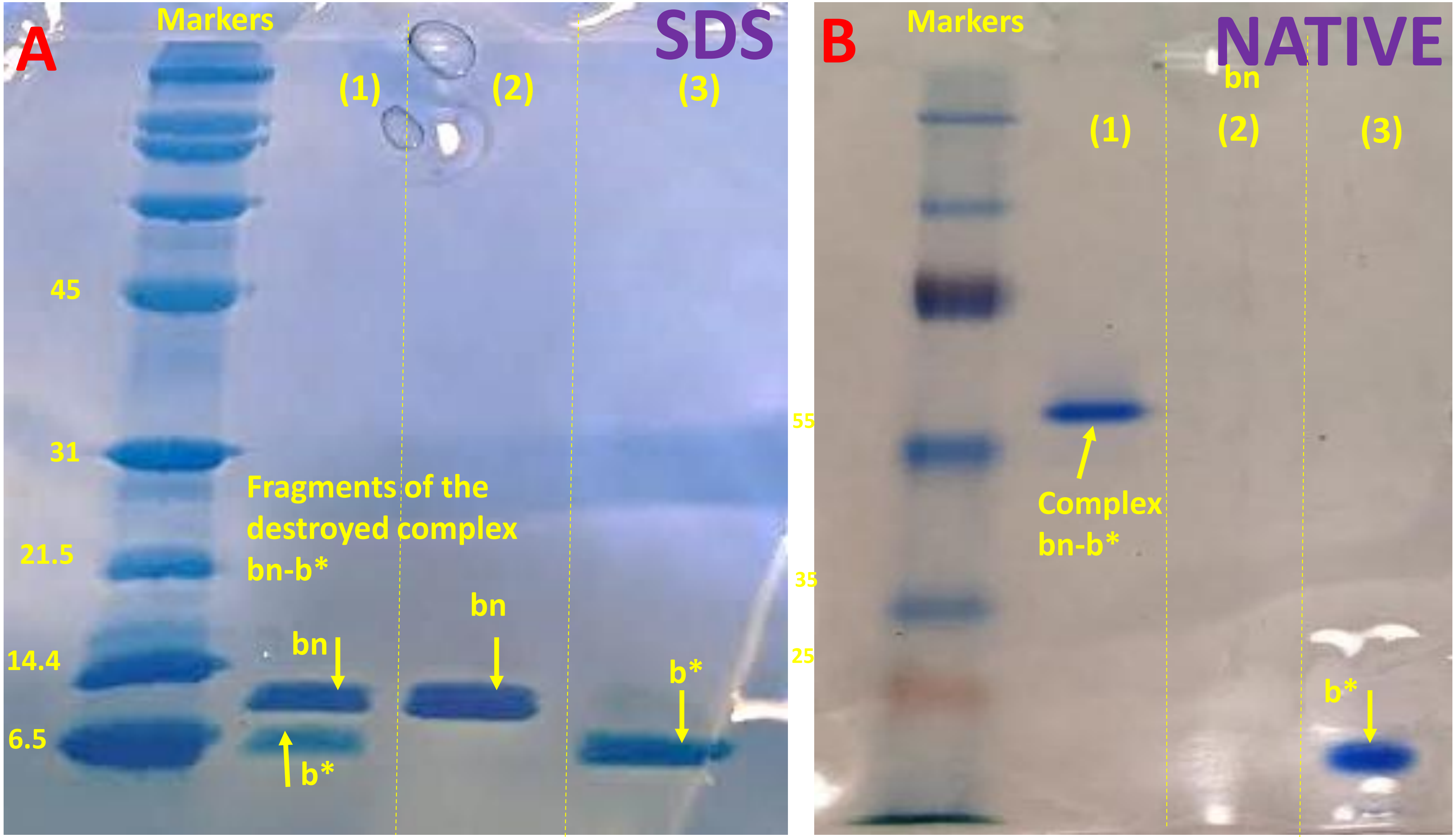

Supplementary Figure S1. The electrophoretic analysis of the barnase-barstar (bn-b*, (1)), barnase (bn, (2)), barstar (b*, (3)) and the mixture in the presence of SDS (A) and in native conditions (B); 12% gel electrophoresis according to Laemli. Molecular weight markers (Bio-Rad 161-0317) 6.5, 14.4, 21.5, 31, 45, 66.2, 97.4, 116.2, and 200 kDa (A) and (Thermo Fisher Scientific 26619) 10, 15, 25, 35, 55, 70, 100, 130, and 250 kDa are shown on the left part of figures.

Protein	Sequence	Length	P	STYCD EHW	KQ N	R	Backbone	Fast exchangeable	Total number of exchangeable hydrogens	Monoisotopic mass
Barnase	AQVINTFDGVADYLQTYHKL PDNYITKSEAQALGWVASKG NLADVAPGKSIGGDIFSNREG KLPGKSGRTWREADINYTSG FRNSDRILYSSDWLIYKTTDH YQTFTKIR	110	3	42	18	6	106	106	212	12375.23
Barstar	MKKAVINGEQIRSISDLHQTL KKELALPEYYGENLDALWD ALTGWVEYPLVLEWRQFEQ SKQLTENGAESVLQVFREAK AEGADITIILS	90	2	31	15	3	87	77	164	10304.29

Supplementary Table S1. Properties of barnase and barstar A. **Length** – protein length in amino acids. **P**, **STYCDEHW**, **KQN**, **R** – number of residues in each of four groups. The amino acids are grouped based on the number of side chain exchangeable hydrogens. **Backbone** – number of exchangeable backbone amides. **Fast exchangeable** - number of exchangeable hydrogens on the side chains (fast exchanging). Calculation were performed using WEB-service http://ms.biomed.cas.cz/MSTools/HDExCalc/HDExCalc.php

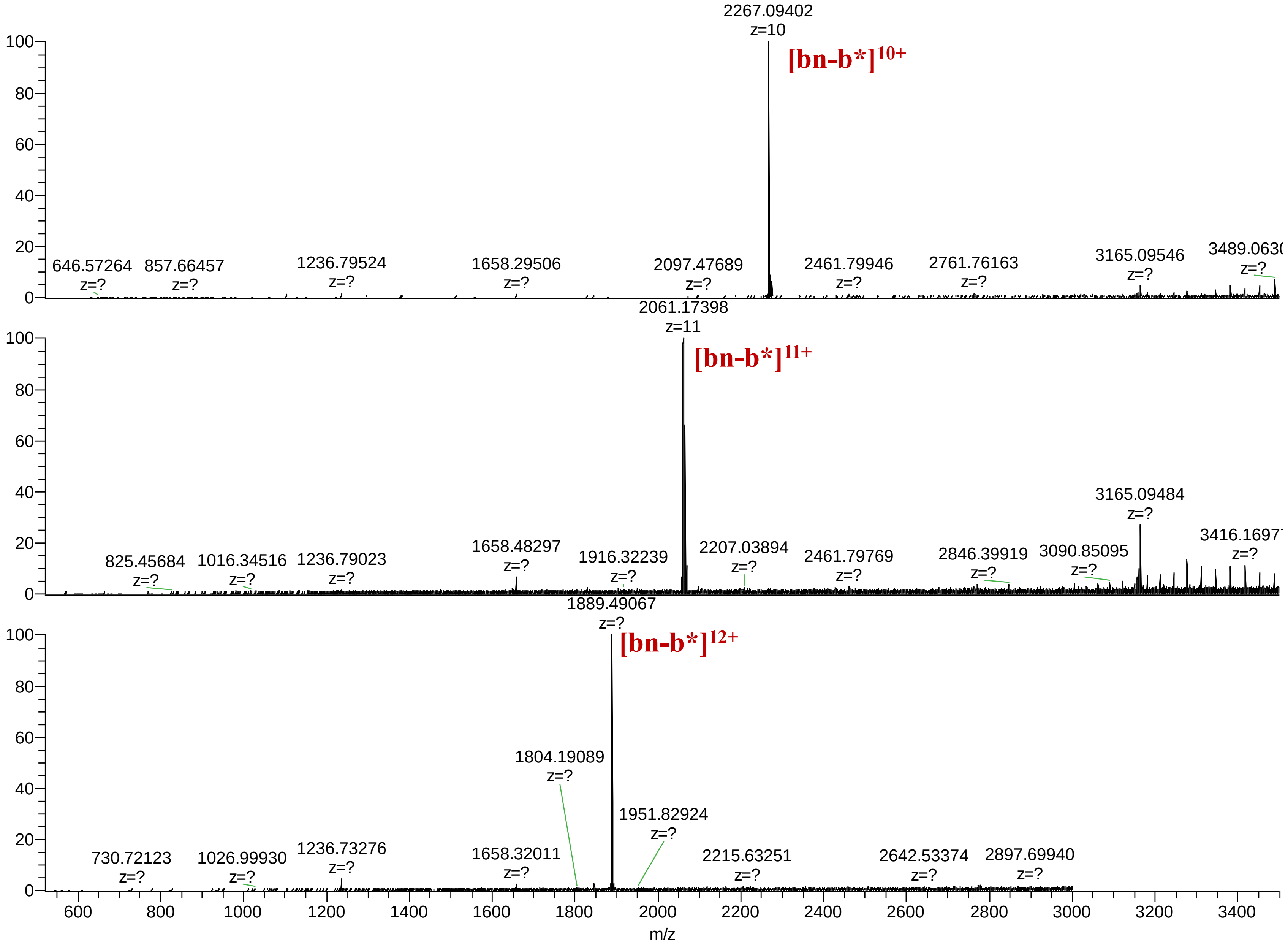

Supplementary Figure S2. Isolation of the bn-b* complex in the ion trap.

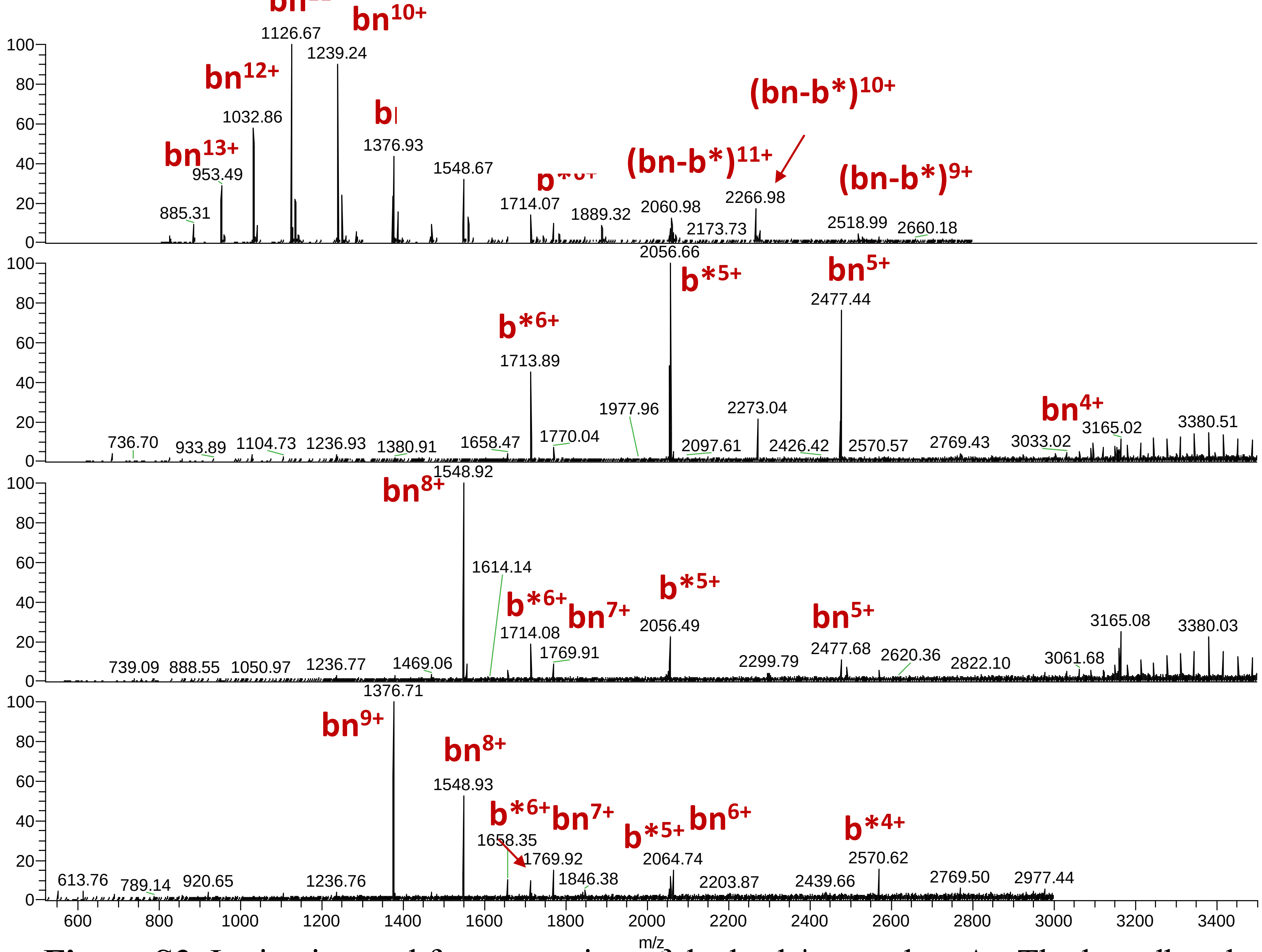

Supplementary Figure S3. Ionization and fragmentation of the bn-b* complex. A - The broadband mass spectrum. B – the fragmentation of the [bn-b*]$^{10+}$, C – the fragmentation of the [bn-b*]$^{11+}$, D – the fragmentation of the [bn-b*]$^{12+}$. The full spectral range.

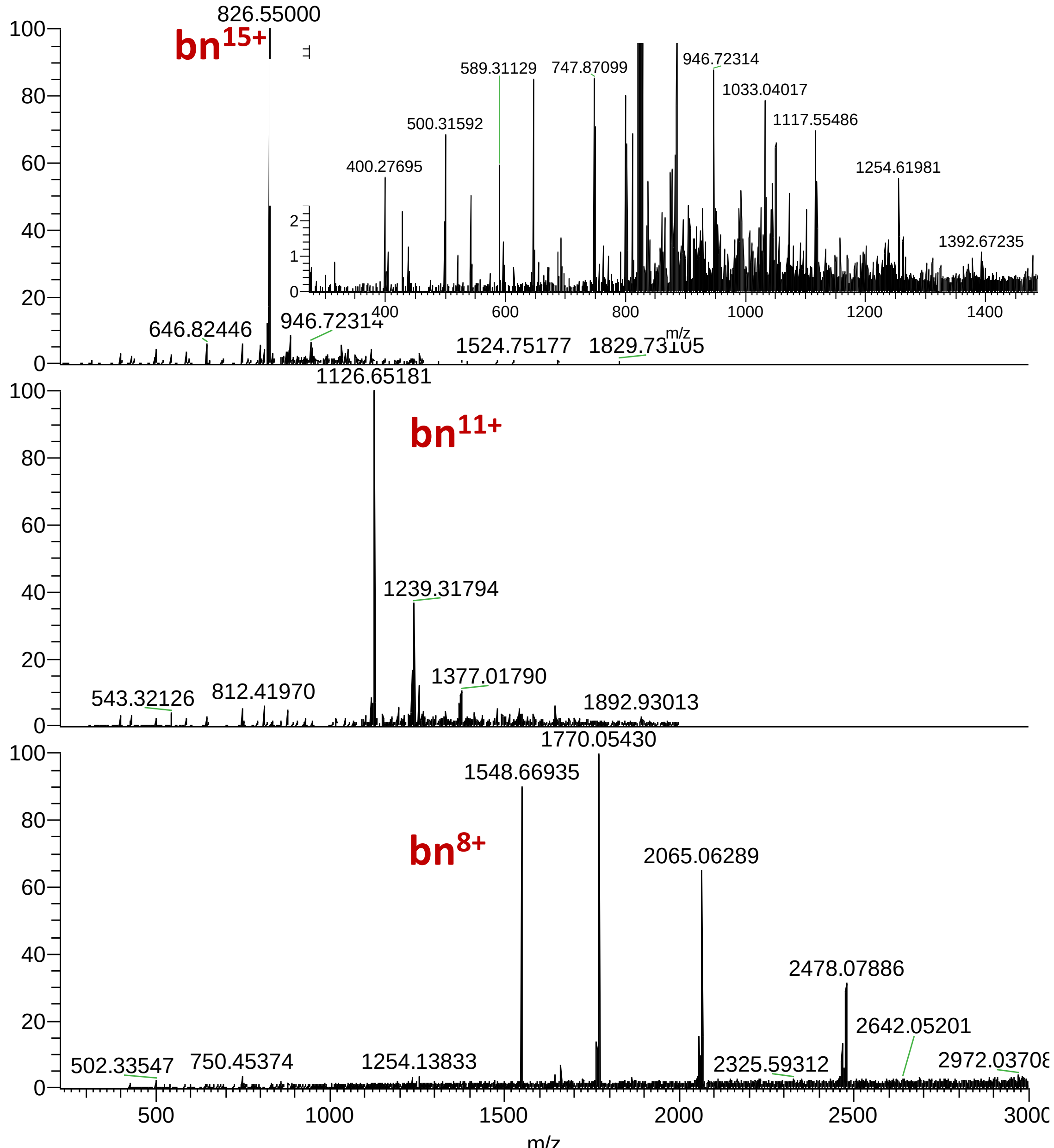

Supplementary Figure S4. The ECD fragmentation of the barnase ion. It can be seen, that only high charge states produce reach fragmentation pattern. Low charge states only capture electron and decrease the charge without the fragmentation.

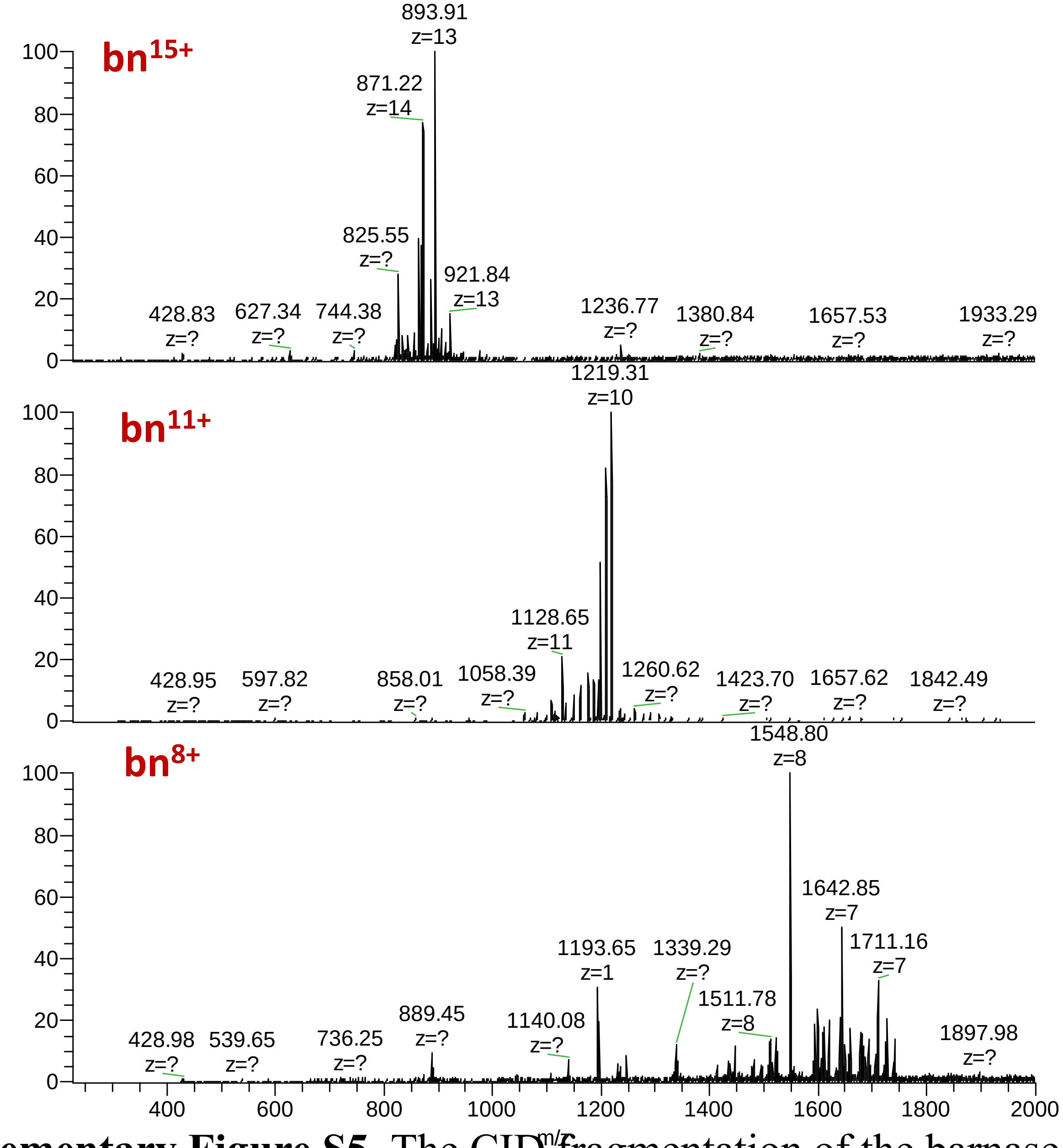

Supplementary Figure S5. The CID fragmentation of the barnase ion. It can be seen, that we didn't obtain reach fragmentation pattern.

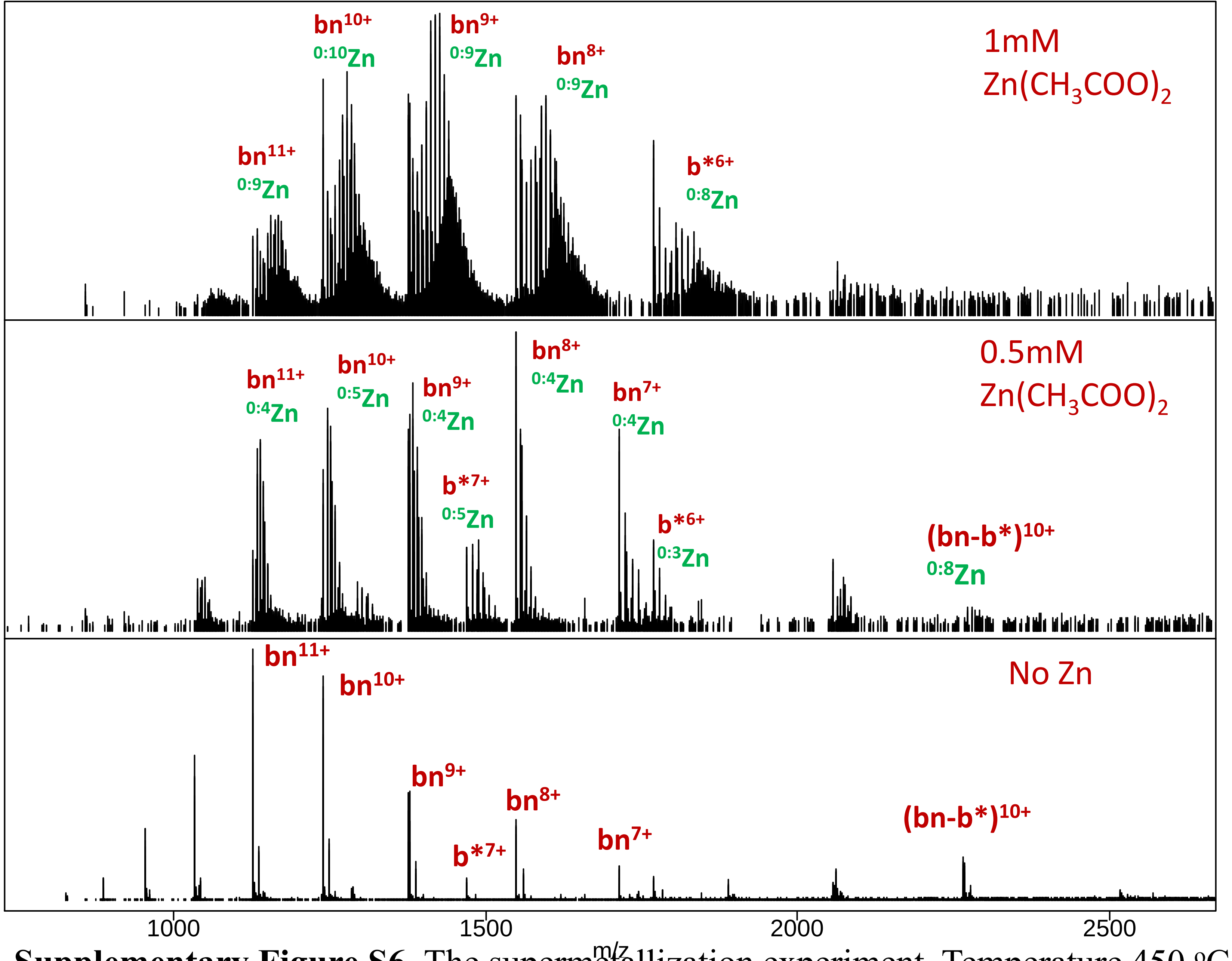

Supplementary Figure S6. The supermetallization experiment. Temperature 450 ^{o}C. The broad band mass spectrum under supermetallization conditions for different concentrations of $Zn(CH_3COO)_2$.
